# Predicting task performance for intelligent human-machine interactions

**DOI:** 10.3389/fnbot.2022.973967

**Published:** 2022-09-13

**Authors:** Jamison Heard, Prakash Baskaran, Julie A. Adams

**Affiliations:** ^1^Adaptive Human-Robot Teaming Lab, Electrical and Microelectornic Engineering Department, Rochester Institute of Technology, Rochester, NY, United States; ^2^Human-Machine Teaming Lab, Robotics Department, Oregon State University, Corvallis, OR, United States

**Keywords:** task performance prediction, human performance modeling, human-machine teaming, deep learning, intelligent system

## Abstract

Human-machine teams are deployed in a diverse range of task environments and paradigms that may have high failure costs (e.g., nuclear power plants). It is critical that the machine team member can interact with the human effectively without reducing task performance. These interactions may be used to manage the human's workload state intelligently, as the overall workload is related to task performance. Intelligent human-machine teaming systems rely on a facet of the human's state to determine how interaction occurs, but typically only consider the human's state at the current time step. Future task performance predictions may be leveraged to determine if adaptations need to occur in order to prevent future performance degradation. An individualized task performance prediction algorithm that relies on a multi-faceted human workload estimate is shown to predict a supervisor's task performance accurately. The analysis varies the prediction time frame (from 0 to 300 s) and compares results to a generalized algorithm.

## 1. Introduction

The NASA control room for the Mars Rover contains a multitude of tasks that must be completed with high performance (Sim et al., 2008) (e.g., generating plans for the robot to complete), as errors may cause significant monetary loss or complete mission failure. Additionally, the human supervisors may experience erratic shift timings, dynamic workload levels (i.e., underload and overload), and fatigue (Leger et al., 2005), which has a substantial negative affect on performance (Wickens et al., 2004). Intelligent human-machine systems seek to optimize human performance in such detrimental conditions by implementing an adaptation strategy (e.g., task autonomy levels) based on human state measurements (e.g., workload, fatigue) (Sim et al., 2008; Sheridan, 2011).

Dynamically allocating task load to the human or system based on cognitive workload is one of the most common adaptation strategies (Kaber and Endsley, 2004; Schwarz and Fuchs, 2018). However, the overall workload is a multi-dimensional construct that is composed of auditory, cognitive, physical, speech, and visual resource channels (McCraken and Aldrich, 1984; Mitchell, 2000). Conflicts can occur between the workload channels (i.e., between auditory and speech) and reduce task performance. More intelligent adaptation strategies can be implemented if the system understands the complete workload state (Heard et al., 2020). For example, if a human's speech workload is overloaded, then an audible alert creates a channel conflict and potentially reduces performance. A system cognizant of the human's speech workload level can postpone the alert or use a visual modality in order to prevent a workload channel conflict from occurring; thus, increasing task performance.

Targeting adaptations to the human's current workload state is a critical component of intelligent human-machine systems, but limits the system's capabilities. There will be a time delay between when an adaptation occurs and when the adaptation affects the human, meaning that an overloaded human may be achieving low task performance before the system's adaptation can mitigate the overloaded workload state. Additionally, a robot or machine's actions must not inadvertently overload the human. A system capable of identifying future task performance decrements or predict an action's potential task load impact on a human may be able to implement an adaptation strategy before an undesired workload state occurs or reason over multiple decisions in order to chose an action that maximizes future task performance.

Consider a wildland fire moving quickly toward a village. An unmanned aerial vehicle (UAS), a remote UAS flight supervisor, and a team of smokejumpers compose the response team. Two overloaded smoke-jumpers are fighting hotspots and cannot be easily replaced. The UAS must determine on which hotspot to drop suppressant. If the UAS relies on current workload state information alone, both hotspots have equal probability of being chosen due to the smokejumper's equal workload levels. However, if the UAS can predict each smokejumper's future task performance and drop suppressant on the hotspot corresponding to the worst performance prediction.

The **research contribution** focuses on a deep-learning model that predicts future task performance using workload estimates corresponding to overall workload and its contributing components (cognitive, physical, visual, auditory, and speech; McCraken and Aldrich, 1984). These workload estimates were provided by a previously developed workload assessment algorithm (Heard and Adams, 2019; Heard et al., 2019) that relied on physiological signals. Using workload estimates as inputs to the performance prediction algorithm created more accurate predictions than when physiological signals were used as inputs. Additionally, relying on workload estimates ensures that the performance prediction algorithm is not constrained to a specific set of physiological signals, which broaden the domains to which it can be applied. For example, consider the NASA control room and wildland fire response scenarios, where electroencephalogram (EEG) data was collected from the control room domain and electrocardiogram (ECG) data was collected from the response domain. If the models rely solely on physiological signals, separate performance prediction models will need to be designed and trained for each domain. Requiring different models is due to the different feature spaces and amount of information in each physiological signal. However, only a single performance model architecture may be needed if a workload assessment algorithm is trained to map the physiological signals to workload estimates in each domain.

Data from a supervisory-based (Scholtz, 2003) human-machine teaming evaluation (Heard et al., 2019) was used to analyze the developed model from two perspectives: *generalized* and *individualized*. The generalized perspective determines how the developed model performs on a human for which it was not trained and is subjected to individual differences. The individualized perspective lessens the impact of individual differences by updating (training) the generalized model with participant-specific information. The **key research contributions** are as follows:

- A deep-learning based task performance prediction algorithm that relies on multi-dimensional workload estimates, rather than solely on physiological signals.
- The algorithm can be generalized across individual users or tailored to an individual human for greater prediction accuracy.
- A robust analysis of the algorithm's ability to predict performance 300 s into the future.

This manuscript is organized as follows: Section 2 reviews related work to performance prediction, while Section 3 describes the methodology for the performance prediction model. The results are presented in Section 4 and discussed in Section 5. The concluding statements are provided in Section 6.

## 2. Related work

Estimating an aspect of the human's state (e.g., workload or engagement) to facilitate intelligent system interactions has been a research focus in a wide range of domains (e.g., adaptive automation, augmented cognition). Adaptive automation systems allocate system control to the human or system in order to mitigate undesired workload states (Kaber and Endsley, 2004; Sheridan, 2011). Similarly, augmented cognition seeks to change task difficulty dynamically in order to keep the human engaged with the system (Fuchs and Schwarz, 2017). Both domains rely on the current human state to determine how to adapt, but generally do not predict the adaptation's impact on task performance. Such a prediction scheme may allow permit more appropriate adaptations.

The current related research relies on physiological measures to indirectly infer task performance, due to the metrics' response to varying workload conditions (Cain, 2007). Ayaz et al. (2019) predicted performance using brain-activity measures (i.e., electroephenogram and functional near-infrared spectroscopy) on a “microscopic” and a “macroscopic” level. The microscopic level predicted performance at most 4 s into the future, which may be too short of a time-frame for an adaptation to prevent a performance decrement from occurring. The macroscopic level predicted performance days into the future, which does not provide information for the current task focus. Other work predicted task performance 15 min into the future using electroephenogram measures (Stikic et al., 2011); however, this work predicted performance using a 10-min block or window. Adaptive systems most likely need a finer granularity of predictions (i.e., 30 s) in order to be effective.

Relying on physiological measures or human state assessments (such as multi-dimensional workload) makes a performance prediction model susceptible to individual differences, as physiological signals vary between individuals (Cain, 2007) and from day to day for the same individual (Christensen et al., 2012). Predicting task performance from human state assessments using generalized and individualized models has yet to be analyzed, but generalized/individualized human state assessments have been developed. Teo et al. (2016, 2019) focused on an individualized workload model for adaptive aiding, by identifying individual specific physiological markers (e.g., EEG and heart-rate metrics) that are sensitive to workload variations. These markers were incorporated in a difference-based workload index algorithm, where individualized algorithms were produced for each human. This approach requires knowing the individual human apriori and it is unclear how the algorithm's internal model may update over time in order to accommodate day-to-day individual differences. A machine-learning based human-state assessment algorithm may incorporate online learning techniques to help account for day-to-day variability (Christensen et al., 2012).

Task performance can be directly measured in some domains (Schreckenghost et al., 2010; McGuire et al., 2019), such as stationary computer-related tasks. However, task performance metrics do not provide the necessary insight into the human's internal state and may hinder future performance predictions. For example, a human can be in an overloaded workload state and achieve high task performance, but the task performance can decrease at some point in the future (Wickens et al., 2004). An adaptive system that relies on workload estimates to infer future performance may detect the overloaded state and reason that task performance will decrease, which can trigger an adaptation to mitigate the decrements.

The developed performance prediction model is applicable across task environments, as the model relies on a multi-dimensional workload estimate, rather than directly using physiological signals. A multidimensional workload estimate is composed of an overall workload continuous value and continuous values for each workload component (cognitive, physical, visual, auditory, and speech). The developed model may be used in a generalized fashion (no apriori human data) or can be individualized to a human teammate for more accurate predictions. The presented performance prediction model differs from the related work as both its generalization and individualization capabilities are analyzed (instead of just one or the other), it relies on continuous multi-dimensional workload estimates (instead of a discrete workload classification), and it is more accurate than solely using physiological signals.

## 3. Methodology

A relationship exists between workload and overall task performance (Wickens et al., 2004); thus, workload information may be used to predict future performance. This information was obtained *via* a workload assessment algorithm (Heard and Adams, 2019; Heard et al., 2019) that estimated the overall workload and each workload component (cognitive, physical, speech, visual, and auditory) every 5 s. This frequency was chosen in order to balance system adaptation rates and workload estimation accuracy (Heard et al., 2020). The algorithm relied on physiological (e.g., heart-rate) data and a 5-layer neural network structure and has been validated in multiple human-machine teaming paradigms (Heard et al., 2019).

A performance prediction model was composed of long short-term memory (LSTM) neural networks (Hochreiter and Schmidhuber, 1997). LSTM networks use previous time-step information to predict future time-steps of a sequential data series. The performance model's input layer consisted of three time-steps (15 s), each with 6 neurons (one neuron for overall workload and each workload component). The input layer was followed by three LSTM layers each with 64 neurons with a dropout rate of 0.8. These layers were followed by a fully-connected layer with 64 neurons and a rectified linear unit activation function. The last layer was an output regression layer with a single linearly activated neuron, which predicted overall performance for *n-timesteps* (the time horizon) in the future. The ADAM optimizer (Kingma and Ba, 2015) with a mean-absolute error (MAE) loss function and a 0.01 initial learning rate was used to train the model. Various other architectures and hyper-parameters were tried, but the presented architecture and respective hyper-parameters correspond to the least complex model that provided comparable performance. Statistical machine-learning approaches (such as a support vector machine) were investigated but did not achieve the desired prediction performance levels.

The entire algorithmic approach is provided in Algorithm 1. First, relevant features are extracted from the physiological data (Line 2) and the features are fed into a workload assessment algorithm (Line 3) to obtain the multi-dimensional workload estimate. It is important to note that including the physiological feature extraction and workload assessment algorithm into the algorithmic approach is necessary to support replicating the methodology, but any physiological signals or workload algorithm may be used. The remaining algorithmic portion is the primary contribution to the performance prediction literature. The current multi-dimensional workload estimate (overall workload and its contributing components) is combined with the prior workload estimates to produce a 1 by 3 (number of timesteps to use) by 6 (number of workload estimates per timestamp) matrix as stated in line 4. This reshaped matrix is fed into a trained LSTM model to predict future task performance (line 5). Any physiological metric or workload assessment algorithm may be used in lines 4 and 5, provided that the workload assessment algorithm can map the physiological metrics to a continuous multi-dimensional workload estimate accurately. Thus, the presented algorithm is not constrained to a specific physiological data set.

**Algorithm 1:**
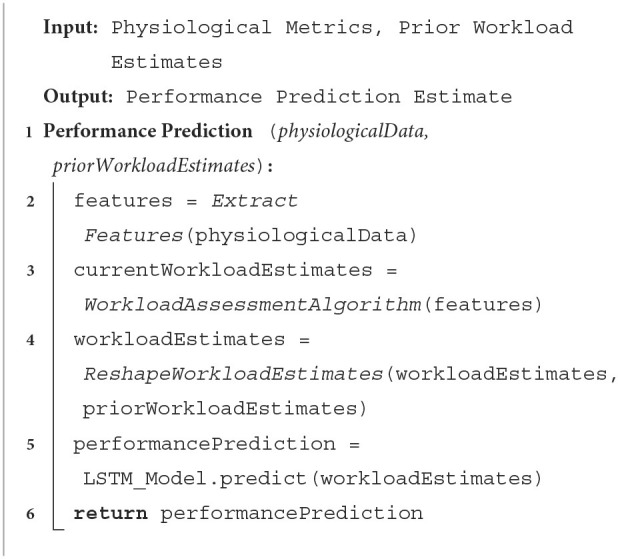
**Performance prediction algorithm**.

### 3.1. Experimental design

Performance data collected from a within-subjects evaluation^1^ (Heard and Adams, 2019; Heard et al., 2019) was used to validate the performance prediction model. Each participant completed 2 days, where the average time between experiment days was 3.48 days (Std. Dev. = 2.13). Each day manipulated workload and measured objective workload, performance, and subjective workload metrics. The first day required each participant to complete a consent form, a demographic questionnaire, and 10-min of training using the NASA MATB-II (Comstock and Arnegard, 1992) prior to completing three 15-min trials. The NASA MATB-II mimics a supervisory human-robot team (Scholtz, 2003), where the human supervises a remotely piloted aircraft. IMPRINT Pro (Archer et al., 2005) was used prior to the evaluation to model the overall workload, its components, and other specifies associated with each trial. The resulting workload models served as the “ground-truth” labels to train the workload assessment algorithm (Heard and Adams, 2019), but were not used for training the performance prediction model.

Each first day 15-min trial corresponded to either the underload (UL), normal load (NL), or overload (OL) workload condition, with trials counterbalanced to negate ordering effects. A 5-min break occurred after the training session and between each first day trial in order to allow the participant's physiological signals to return to their resting state levels (Reimer et al., 2009). The second day emulated real-world conditions in which workload transitioned between levels (e.g., UL to OL to NL). Each participant completed one 35-min trial, which contained seven consecutive 5-min workload conditions. The three workload condition orderings were as follows:

- **O_1_**: UL-NL-OL-UL-OL-NL-UL
- **O_2_**: NL-OL-UL-OL-NL-UL-NL
- **O_3_**: OL-UL-OL-NL-UL-NL-OL

The orderings were chosen, such that each workload condition transition occurred once. The second day workload conditions were not randomized, given the focus on workload transitions, rather than the conditions themselves. Additionally, the 5-min time-frame per condition reflects the time that physiological signals need to identify a workload transition (Reimer et al., 2009). Multiple works have investigated physiological responses to various workload states (Castor, 2003; Cain, 2007; Heard, 2019). For example, there is typically an increase in heart-rate when transitioning from a lower workload state to a higher workload state. Vice-versa, heart-rate decreases when transitioning to a lower workload state.

**Figure 1:**
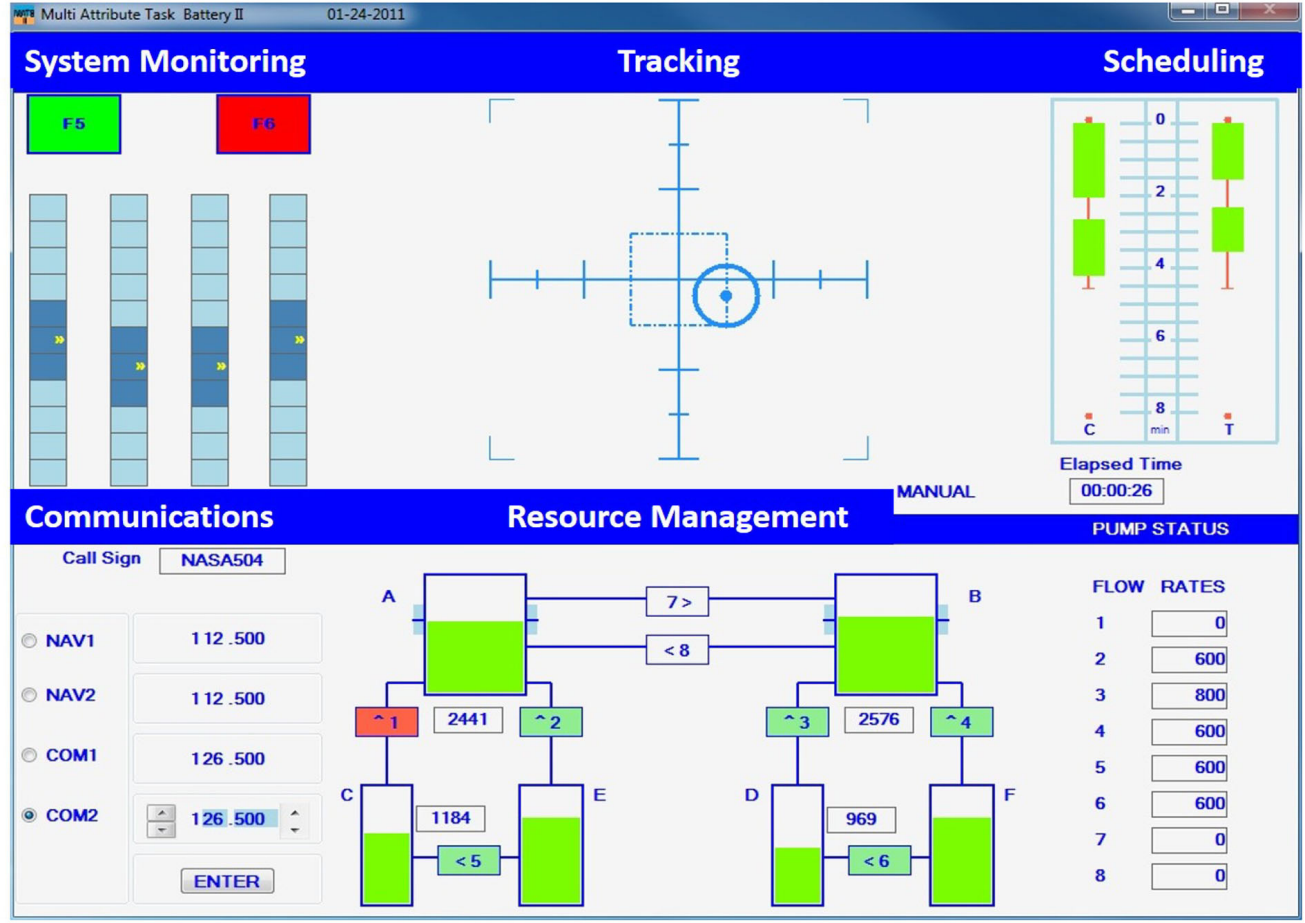
The NASA MATB-II task environment.

The evaluation's task environment consisted of the NASA MATB-II, which simulated a human supervising a remotely-piloted aircraft. The simulation, depicted in Figure 1, incorporated four concurrent tasks: tracking, system management, resource management, and communication monitoring. Each task elicits different demand levels (e.g., cognitive, visual, physical), which was captured in the IMPRINT Pro software (Heard, 2019). Participants also have differing skill levels, where some tasks may be easier than others (e.g., the tracking task may be easier for someone with prior flight simulator experience). This varying skill level will impact overall performance; therefore, individual-specific performance prediction models are needed.

Participants were to maintain a blue circle within the center of the crosshairs using a joystick for the tracking task, see the top, center of Figure 1. This task operated in two modes (automatic and manual) that were used for manipulating workload. The automatic mode required no participant input and was used for the entire duration of the underload condition. The manual mode required the participant's full control and was used throughout the overload condition. The modes switched approximately every 2.5 min during the normal workload condition, as determined by the IMPRINT Pro workload modeling.

The system monitoring tasks' two colored buttons and four gauges are depicted in the upper left of Figure 1. This task requires identifying when a button or a gauge was out of range and resetting it by selecting the element. The green button (F5) was out of range when it turned off (gray), while the red button (F6) was out of range when turned on (red). A randomly moving indicator (up and down) typically remained in the middle of each gauge. If the indicator was too high or too low, the gauge was to be clicked on in order to reset it. The underload condition had one out of range instance per minute, overload had twenty instances per minute, and normal load had five instances per minute.

Six fuel tanks (A-F) and eight fuel pumps (1-8) were monitored for the resource management task, depicted in the bottom center of Figure 1. The fuel levels of Tanks A and B were to be maintained between 2,000 and 3,000 units by turning fuel pumps on or off, where the arrow depicted fuel direction. Fuel Tanks C and D had finite fuel levels, while Tanks E and F had an infinite fuel supply. Workload was manipulated by the number of failed pumps, indicated by the pump turning red and zero fuel flowing. Zero pumps failed during the underload condition, while two or more pumps failed during the overload condition. The normal load condition switched from zero pumps failing to one or two pumps failing every minute.

The communications task required listening to air-traffic control requests for radio changes. A request may be “NASA 504, please change your COM 1 radio to frequency 127.550.” The original MATB-II communications task required no speech; thus, a required verbal response was added. A response may be “This is NASA 504 tuning my COM 1 radio to frequency 127.550.” Participants were to change the specified radio to the specified frequency by selecting the desired radio and using the arrows to change the radio's frequency, as depicted in the lower left of Figure 1. Communications not directed to the participant, as indicated by the call sign, were to be ignored. The underload condition contained two or fewer requests, the overload contained eight or more, and the normal load contained from two to eight requests per minute.

### 3.2. NASA MATB-II overall task performance

The performance prediction model requires overall task performance data in order to be validated, but no current method exists to combine each NASA MATB-II task performance measure into an overall measure. The individual performance measures were mapped to a value from 0 to 1, where 1 represents optimal performance. The tracking task performance measure (i.e., root-mean squared error between the crosshairs' center and the object) was normalized based on participant data. The system monitoring task and communications task performances were measured using two metrics: reaction time and success rate. *Reaction time* represents the time a participant took to correct an out-of-range light or gauge, while *success rate* represents the number of out-of-range instances corrected divided by the total number of instances. Reaction time was normalized to the range 0–1, while success rate was already within range (0–1). A value of 1 was assigned if the resource management task's fuel levels were within 2,000 and 3,000 units, while the tank levels were normalized (0–1) outside of that range. The *overall performance measure* was the uniform average of all active tasks' performance measures, which assume that the tasks' trade offs will be equivalent in terms of performance. For example, if the tracking and system monitoring tasks were the only active tasks, then the overall performance was the average of those tasks' measures.

Relying on normalizing the performance metrics and using an uniform average calculation may not be the optimal solution to generating an overall performance score. Normalizing performance data does not penalize time dependent performance measures. For example, the fuel tank levels rise at a constant rate; thus, fuel levels much smaller than 2,000 units were penalized more than fuel levels close to 2,000 units. Developing appropriate time penalizations is not trivial and is tangential to the developed model's performance. Using a uniform average to calculate overall task performance does not account for task priority levels, but the participants were not given task prioritizations; thus, the use of a uniform average.

### 3.3. Participants

The thirty participants (18 female and 12 male) had a mean age of 25.70 [Standard Deviation (St. Dev.) = 8.65], with an age range from 18 to 62. Caffeine usage and an individual's fitness may impact the physiological metrics' response to workload. Seventeen participants did not drink any caffeine the day of the experiment, while six drank ≤ 16 oz and seven drank ≥ 16 oz. Participants indicated that they exercised a mean of 3.86 (St. Dev. = 1.59) times per week. Video game experience was also collected, where 25 participants played video games for three or fewer hours per week.

The participants slept an average of 6.58 (St. Dev. = 1.57) h the night before the first day of the experiment and an average of 6.78 (St. Dev. = 1.85) h two nights prior. The participants' stress levels, rated on a Likert scale (1-little to 9-extreme), were rated as 2.90 (St. Dev. = 1.76), while fatigue levels were rated as 2.83 (St. Dev. = 1.32) on the same scale.

### 3.4. Metrics

Objective and subjective workload measures were collected throughout the evaluation. The BioPac BioHarness™3 portable measurement device was attached to a flexible strap that fastens around the participants' ribs against the skin, much like an athletic heart rate monitor. This device captured the heart rate, heart-rate variability, respiration rate, skin temperature, body activity, and posture objective workload metrics. The other objective workload metrics, such as noise level and speech rate, were collected using a REED R8080 decibel meter and a Shure PGX1 microphone head set, respectively.

The subjective workload measures consisted of verbal *in situ* workload ratings (Harriott et al., 2013) and the NASA-TLX (Hart and Staveland, 1988). The verbal ratings were administered every four and a half min, while the NASA-TLX was completed after each trial.

### 3.5. Validation methodology and hypotheses

The remainder of this paper validates the performance prediction models' ability to accurately predict and track the task performance trends within and across workload conditions from two perspectives: *generalized* and *individualized*. The *generalized* perspective determined how the prediction model performs on an unseen human (i.e., generalizes across a population), by using a leave-one-participant-out cross-validation scheme. The *generalized* model was trained on 29 participants' performance data from both evaluation days and tested on the corresponding second day data of the remaining (left-out) participant. This scheme was repeated 30 times, once for each participant.

Higher predictive power may be achieved using data from a specific individual; thus, the *individualized* perspective updated the previously trained *generalized* model (i.e., 29 participants data) by training the model with the remaining participant's data (workload estimates) from the first evaluation day and the first half of the second day. The *individualized* model was tested on the remaining second day's data. This validation scheme simulates a potential real-world application, where a *generalized* performance prediction model is developed for all of the human supervisors. This *generalized* model can later be tailored to an individual during training (the evaluation's first day) and from day-to-day use (the evaluation's second day). The *generalized* and *individualized* model both use multi-dimensional workload estimates (overall workload and each workload component) as inputs. No individual demographic data were used.

Both models were compared to a respective physiological-signal only model, which used the same features that the workload assessment algorithm relied on and the same LSTM model architecture. This comparison is to demonstrate that relying on workload estimates can provide more accurate performance predictions than relying solely on physiological-based features. The training and validation scheme for analyzing the physiological-based model was the same as the workload estimate-based model.

Several hypotheses were developed. Hypothesis **H**_**1**_ predicted that the MAE between the actual and the predicted performance values will be less than 0.1 for the generalized model. This value was the difference between the measured average participant overall performance values from each evaluation workload condition: UL 0.91 (St. Dev. = 0.17), NL 0.82 (St. Dev. = 0.18), and OL 0.72 (St. Dev. = 0.13). Pearson's Correlation Coefficient was used to analyze the prediction model's ability to track performance trends, both within and across workload conditions. Hypothesis **H**_**2**_ predicted that the generalized models' predictions will correlate positively and significantly with the measured values.

The individualized performance prediction models' estimates were expected to track the performance trends better than the corresponding generalized models; therefore, hypothesis **H**_**3**_ predicted that the individualized performance prediction model's MAE values will be less than the corresponding generalized model. Similarly, hypothesis **H**_**4**_ predicted that the individualized model's Pearson correlation coefficients will be larger than the generalized model's coefficients.

The predictive power of both the generalized and individualized performance prediction models was evaluated by developing seven prediction variants for each model. These prediction variants predicted the task performance at various time horizons (future timesteps in seconds): 0 s (i.e., current performance), 5, 15, 30, 60, 120, and 300 s. These time horizons were chosen in order to capture the expected trade-off between time horizon and accuracy. It was expected that predicting performance past 300 s into the future will be an inadequate time-frame for system adaptations, due to the stationary nature of time-series signals. The predictive power of both the *generalized* and *individualized* performance prediction models was expected to decrease with the increase in the time horizon. Hypothesis **H**_**5**_ predicted that the MAE values will increase, and the correlation coefficients will decrease with the increase in the time horizon.

Lastly, it is expected that using workload estimates, rather than physiological-based features, will result in more accurate predictions. Specifically, hypothesis **H**_**6**_ predicts that MAE's will be lower and the correlation coefficients will be higher using workload estimates as inputs, than using physiological-based features.

## 4. Results

The following results correspond to the two performance prediction models: *generalized* and *individualized*. The *generalized* model's results pertain to the described leave-one participant out cross validation scheme, which was used to determine the impact of individual differences. The *individualized* model's results lessen the impact of individual differences, by training the *generalized* model with individual-specific data.

### 4.1. Generalized performance prediction model

Seven prediction model variants were developed for the *generalized* performance prediction model: **GM**_**0**_**, GM**_**5**_**, GM**_**15**_**, GM**_**30**_**, GM**_**60**_**, GM**_**120**_, and **GM**_**300**_. The **GM**_**0**_ model variant predicted a participant's current task performance, while the **GM**_**5**_, **GM**_**15**_, **GM**_**30**_, **GM**_**60**_, **GM**_**120**_, and **GM**_**300**_ variants predicted the task performance for 5, 15, 30, 60, 120, and 300 s, respectively, into the future.

Mean absolute error measures the absolute error between the generalized model's predicted values and the actual (measured) participant performance values. The MAE for each of the evaluation's second day's workload orderings (i.e., **O_1_**, **O_2_**, and **O_3_**) are provided in Table 1 by workload conditions. The **All** column presents the aggregate MAE (the error across the workload conditions). Overall, the worst MAE was 0.162 (Order 1 NL), which means that the corresponding model prediction is within 0.162 of the actual participant performance (ranged [0, 1]). For example, if a predicted performance value was 0.8, the actual performance value is within the range 0.64–0.96. Likewise, the best MAE was 0.064 (Order 3 UL), which changes the actual performance range to 0.73–0.87, given a predicted value of 0.8.

**Table 1:** The predicted performance mean absolute error (MAE) for each generalized model variant by workload condition and ordering.

**Ordering**	**Model**	**UL**	**NL**	**OL**	**All**
**O_1_**: UL-NL-OL-UL-OL-NL-UL	GM_0_	**0.117**	**0.158**	0.111	**0.131**
	GM_5_	**0.117**	0.162	**0.110**	0.133
	GM_15_	0.120	0.162	0.116	0.136
	GM_30_	0.126	0.161	0.117	0.137
	GM_60_	0.132	0.163	0.119	0.141
	GM_120_	0.142	0.162	0.130	0.147
	GM_300_	0.153	0.159	0.126	0.147
**O_2_**: NL-OL-UL-OL-NL-UL-NL	GM_0_	0.099	**0.134**	**0.077**	**0.107**
	GM_5_	0.100	**0.134**	0.081	0.109
	GM_15_	0.102	0.135	0.082	0.110
	GM_30_	0.099	**0.134**	0.091	0.111
	GM_60_	0.094	0.138	0.101	0.113
	GM_120_	**0.090**	0.142	0.125	0.120
	GM_300_	0.094	0.145	0.125	0.119
**O_3_**: OL-UL-OL-NL-UL-NL-OL	GM_0_	**0.064**	0.115	0.100	**0.094**
	GM_5_	0.065	**0.114**	**0.099**	**0.094**
	GM_15_	0.069	0.116	0.101	0.097
	GM_30_	0.073	0.115	0.106	0.099
	GM_60_	0.066	0.114	0.114	0.099
	GM_120_	0.072	0.115	0.128	0.105
	GM_300_	0.071	0.115	0.108	0.098

The lowest values for each workload condition are in **bold**.

Each model variant's MAE in the UL and OL conditions for **O_2_** and **O_3_** were below 0.10, indicating a 0.2 range around a predicted value (e.g., 0.7–0.9 for a predicted value of 0.8). The general model variants had more difficulty with Order **O_1_** and predicting performance during the normal load condition (MAE above 0.1). This result indicates that ordering effects occurred. Additionally, the higher error for the normal load condition is attributed to the large range of workload values compared to the underload and overload conditions.

The models' ability to track performance trends, both within and across workload conditions was analyzed using Pearson's correlations, where a larger positive coefficient represents better tracking. The correlation coefficients are presented in Table 2. Almost all of the correlations across the workload conditions were positive and significant; however, these correlation coefficients were all ≤ 0.5 (moderate positive relationship, rather than a strong positive relationship). The GM_0_, GM_5_, GM_15_, and GM_30_ prediction models' estimates were more positively correlated with the actual performance values than those with longer time horizons.

**Table 2:** The Pearson's correlations between the predicted performance and actual performance for each generalized model by workload condition and each workload ordering.

**Ordering**	**Model**	**UL**	**NL**	**OL**	**All**
**O_1_**: UL-NL-OL-UL-OL-NL-UL	GM_0_	0.333**	0.071*	-0.004	0.211**
	GM_5_	0.325**	-0.019	**0.066**	0.188**
	GM_15_	0.255**	-0.057	-0.123**	0.138**
	GM_30_	0.226**	0.007	-0.059	0.142**
	GM_60_	0.179**	0.038	0.060	0.136**
	GM_120_	-0.096**	-0.058	-0.078	-0.049*
	GM_300_	0.038	-0.097**	-0.122**	-0.036
**O_2_**: NL-OL-UL-OL-NL-UL-NL	GM_0_	0.186**	0.096**	0.582**	0.291**
	GM_5_	0.195**	0.184**	0.560**	0.306**
	GM_15_	0.174**	0.167**	0.445**	0.302**
	GM_30_	0.242**	0.184**	0.230**	0.300**
	GM_60_	0.315**	0.147**	0.290**	0.300**
	GM_120_	0.262**	0.161**	0.207**	0.259**
	GM_300_	0.205**	0.214**	-0.065*	0.213**
**O_3_**: OL-UL-OL-NL-UL-NL-OL	GM_0_	0.359**	0.116**	0.523**	0.485**
	GM_5_	0.333**	0.145**	0.504**	0.486**
	GM_15_	0.237**	0.094**	0.472**	0.458**
	GM_30_	0.192**	0.112**	0.388**	0.440**
	GM_60_	0.188**	0.188**	0.212**	0.388**
	GM_120_	0.109**	0.196**	0.271**	0.295**
	GM_300_	0.277**	0.053	0.348**	0.266**

*Indicates *p* ≤ 0.05 and **indicates *p* ≤ 0.01. The highest values for each workload condition are in **bold**.

Examining the average change in MAE and the correlation coefficient as the time horizon increases permits determining at what time horizon the algorithmic performance degrades. The MAEs typically increased on average 0.002 as the time horizons increased, which indicates a minimal change in error when predicting further into the future. This minimal change was unexpected, as it was expected to have more inaccurate predictions further into the future. However, the correlation coefficients decreased on average by 0.03 with an increase in the time horizon, where this large change was expected. Overall, the average error for longer time horizons changes minimally, but the model variants are unable to track the overall performance trends.

**Figure 2:**
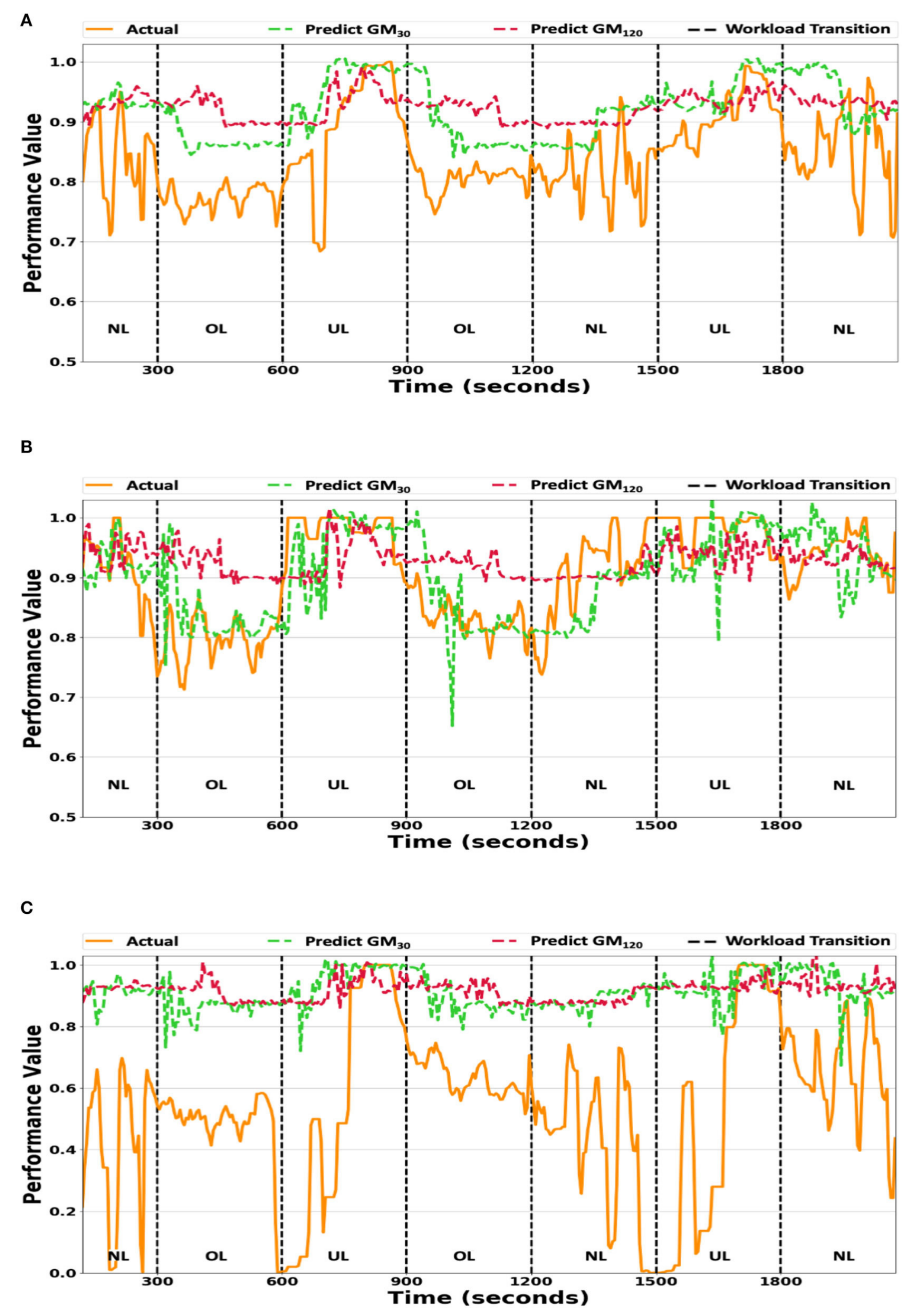
**(A)** The generalized model's predictions vs. the averaged actual participant performance values. **(B)** The generalized model predictions vs. the true performance values of participant P_*best*_. **(C)** The generalized model predictions vs. the true performance values of participant P_*worst*_. The GM_30_ and GM_120_ prediction model variant's predicted values plotted against the true performance values for workload ordering **O_2_**. The y-axis scale ranges from 0.5 to 1.0 for **(A,B)**, while it ranges from 0.0 to 1.0 for **(C)**. **UL/NL/OL** in each plot represents the workload condition.

The GM_30_ and GM_120_ predicted and actual performance values for **O_2_** were plotted in Figure 2 since they are the most likely to be used in an intelligent human-machine system and **O_2_** contains the most instances of the NL condition (the highest variance in performance, as stated in Section 3.5). GM_60_ may also be used in an intelligent human-machine system; however, the model's estimates are similar to GM_30_ and incorporating the estimates in the plot makes the figure too cluttered. Figure 2A plots the average **O_2_** participant performance at each timestep. The two *generalized* model variants overestimated the actual average participant performance, but tracked the high-level trends (i.e., performance decreasing/increasing across workload conditions). Low-level trends (i.e., within a workload condition) were not tracked well by either prediction model, as evident during the first NL condition (0–300 s).

The *generalized* model's accuracy for two participants, whose performance models' MAE were the lowest (P_*best*_) and highest (P_*worst*_), are presented in Figures 2B,C. The GM_30_ and GM_120_ model variants failed to track small fluctuations in the actual individual participants' performance values, as seen during the first OL condition (300–600 s). The GM_30_ model variant tracked a large increase/decrease in the actual performance values with an approximate time delay of 10–20 s, as depicted during the workload transition at 600 s in Figure 2B. The GM_120_ model's response was either flat or noisy and did not correlate well with the actual individual participant performance values. Additionally, there is little variance between P_*best*_'s (Figure 2B) and P_*worst*_'s (Figure 2C) model estimates, indicating the *generalized* model fits the average participant performance, but does not capture individual participant trends well.

### 4.2. Individualized performance prediction model

Similar to the generalized prediction methodology, the predictive power of the *individualized* performance prediction model was evaluated by developing the seven prediction model variants: **IM**_**0**_**, IM**_**5**_**, IM**_**15**_**, IM**_**30**_**, IM**_**60**_**, IM**_**120**_, and **IM**_**300**_. The MAE for each *individualized* model variant by workload ordering and workload condition are provided in Table 3, where **bold** values represent an increase in accuracy over the corresponding GM model. Results for **O_2_**'s OL condition cannot be presented for the IM_300_ variant, because that condition only occurs at the beginning of the second half of the evaluation's second day. Thus, predicting the performance 300 s into the future caused the IM_300_ model variant to avoid the OL condition. Overall, there are no clear trends in the table. However, the IM model variants on average had an 0.02 decrease in MAE from the corresponding GM MAEs (Table 1), demonstrating that individual specific information improved model performance. There was an average increase in MAE of 0.005 for **O_3_**'s UL condition. This marginal increase was unexpected but does not substantially affect the overall prediction results.

**Table 3:** The predicted performance MAE for each individualized model by workload condition and workload ordering.

**Ordering**	**Model**	**UL**	**NL**	**OL**	**All**
**O_1_**: UL-NL-OL-UL-OL-NL-UL	IM_0_	**0.110**	**0.136**	**0.052**	**0.106**
	IM_5_	0.119	**0.145**	**0.054**	**0.112**
	IM_15_	**0.118**	**0.135**	**0.052**	**0.108**
	IM_30_	**0.122**	**0.139**	**0.057**	**0.112**
	IM_60_	**0.129**	**0.140**	**0.063**	**0.116**
	IM_120_	**0.126**	**0.139**	**0.080**	**0.118**
	IM_300_	**0.147**	**0.126**	**0.075**	**0.117**
**O_2_**: NL-OL-UL-OL-NL-UL-NL	IM_0_	**0.086**	**0.117**	**0.063**	**0.093**
	IM_5_	**0.089**	**0.117**	**0.062**	**0.094**
	IM_15_	**0.090**	**0.114**	**0.055**	**0.091**
	IM_30_	**0.088**	**0.115**	**0.059**	**0.093**
	IM_60_	**0.079**	**0.115**	**0.055**	**0.089**
	IM_120_	**0.088**	**0.116**	**0.057**	**0.095**
	IM_300_	**0.085**	**0.107**	N/A	**0.097**
**O_3_**: OL-UL-OL-NL-UL-NL-OL	IM_0_	0.068	**0.092**	**0.068**	**0.082**
	IM_5_	0.071	**0.092**	**0.070**	**0.084**
	IM_15_	0.071	**0.096**	**0.064**	**0.085**
	IM_30_	0.078	**0.095**	**0.062**	**0.086**
	IM_60_	0.070	**0.090**	**0.071**	**0.082**
	IM_120_	0.080	**0.087**	**0.070**	**0.083**
	IM_300_	0.079	**0.090**	**0.069**	**0.084**

The **bold** MAEs are lower than the corresponding GM model's MAEs.

The *individualized* models' correlations with the actual performance values are presented in Table 4. Most of the correlations were positive and significant for each workload condition and ordering. Similar to the MAE results, the correlations were on average 0.36 higher than the corresponding generalized model's correlations, except for **O_3_**'s UL condition. The individualized models' correlations were the highest during the OL condition for all three workload orderings.

**Table 4:** The Pearson's correlations between the predicted performance and actual performance for each individualized model variant by workload condition and ordering.

**Ordering**	**Model**	**UL**	**NL**	**OL**	**All**
**O_1_**: UL-NL-OL-UL-OL-NL-UL	IM_0_	0.751**	0.731**	0.926**	0.689**
	IM_5_	0.822**	0.701**	0.907**	0.656**
	IM_15_	0.712**	0.753**	0.877**	0.693**
	IM_30_	0.743**	0.763**	0.842**	0.689**
	IM_60_	0.677**	0.712**	0.817**	0.672*
	IM_120_	0.598**	0.744**	0.761**	0.681**
	IM_300_	0.576**	0.727**	0.699**	0.683**
**O_2_**: NL-OL-UL-OL-NL-UL-NL	IM_0_	0.414**	0.722**	0.828**	0.662**
	IM_5_	0.387**	0.734**	0.831**	0.648**
	IM_15_	0.396**	0.730**	0.851**	0.654**
	IM_30_	0.424**	0.708**	0.845**	0.653**
	IM_60_	0.468**	0.728**	0.846**	0.684**
	IM_120_	0.429**	0.751**	0.832**	0.680**
	IM_300_	0.464**	0.738**	N/A	0.627**
**O_3_**: OL-UL-OL-NL-UL-NL-OL	IM_0_	0.318**	0.506**	0.879**	0.557**
	IM_5_	0.228**	0.545**	0.872**	0.564**
	IM_15_	0.207**	0.477**	0.843**	0.527**
	IM_30_	0.009	0.485**	0.796**	0.510**
	IM_60_	0.082	0.524**	0.647**	0.495**
	IM_120_	0.146**	0.576**	0.739**	0.478**
	IM_300_	0.348**	0.723**	0.819**	0.590**

*Indicates *p* ≤ 0.05 and **indicates *p* ≤ 0.01. The **bold** correlations are higher than the corresponding GM models' correlations.

Similar to the GM model results, there was a negligible change in MAE as the time horizon increased and very low overall variance (0.0006) across the values. An average decrease of 0.004 in the correlation coefficients existed, which is much lower than the 0.03 decrease for the GM model variants. This result indicates that individual models are better able to track performance trends, than the corresponding GM models, as the time horizon increases.

**Figure 3:**
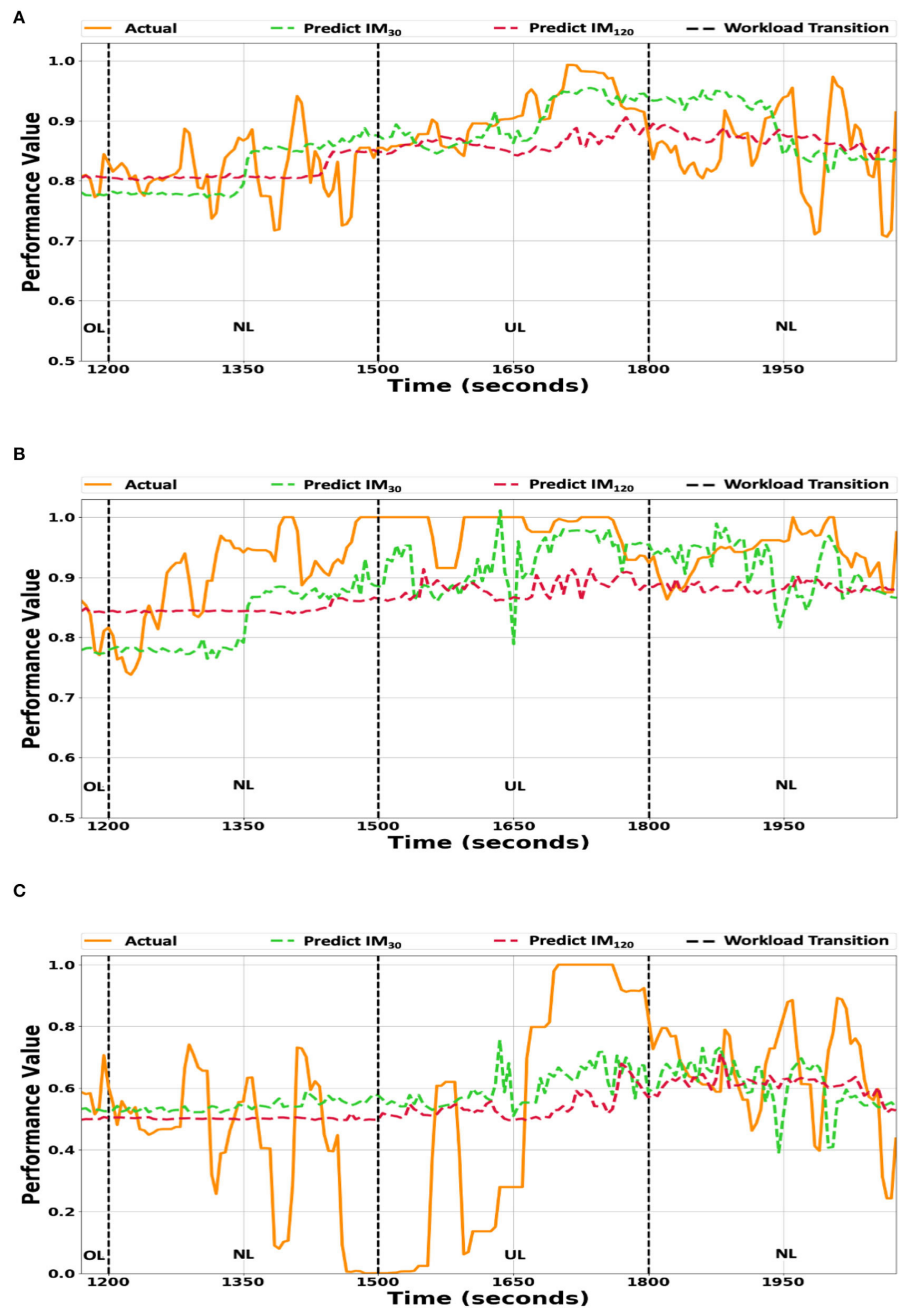
**(A)** The individualized model predictions vs. the averaged participants' true performance values. **(B)** The individualized model predictions vs. the true performance values of participant P_*best*_. **(C)** The individualized model predictions vs. the true performance values of participant P_*worst*_. The IM_30_ and IM_120_ prediction model variant's predictions plotted against the true performance values for workload ordering **O_2_**. The y-axis scale ranges from 0.5 to 1.0 for **(A,B)**, while for **(C)**, it ranges from 0.0 to 1.0. **UL/NL/OL** in the plots represent the workload condition.

The IM_30_, and IM_120_ performance values and the actual performance values for the **O_2_** workload ordering were plotted in Figure 3, while the IM model estimates vs. the average participant performance values were plotted in Figure 3A. The performance predictions and the actual participant performance for both *individualized* model variants were within 0.1 of each other. The IM_30_ model predictions tracked the workload transitions better than the IM_120_ model predictions. The two model variants had difficulty tracking quick transitions, which can be seen during the first NL condition (0–300 s) in Figure 3A. The individualized models tracked the actual performance values during workload condition transitioning (i.e., at 1,200, 1,500, and 1,800 s) better than the *generalized* models (Figure 2A).

The IM_30_ and IM_120_ model predictions for participants P_*best*_ and P_*worst*_ are depicted in Figures 3B,C, respectively. The IM_30_ and IM_120_ model variants' predictions tended to underestimate P_*best*_'s actual performance (Figure 3B), which may be attributed to the bias present in the *individualized* models caused by retraining the *generalized* model. The *individualized* model variants' predictions for Participant for Participant P_*worst*_'s had difficulty capturing the large variance in the actual performance values, which can be seen between 1,200 s and 1,500 s in Figure 3C. The two *individualized* model variants' predictions were closer to the actual performance values than the corresponding *generalized* model variants (Figure 2).

### 4.3. Comparison to physiological-based model

The developed performance prediction model relies on multi-dimensional workload estimates in order to create a more flexible approach to performance prediction. However, there may be some utility in relying solely on physiological signals. Thus, an analysis was performed to determine accuracy differences between the developed workload estimate-based model and a physiological-based model.

This comparison analysis only considers accuracy on the entire testing set, rather than individual workload conditions and orderings. Table 5 presents the overall MAEs and correlation coefficients by model and input type. Overall, the workload estimate-based model results in lower MAEs than the respective physiological-based model. However, the difference in MAE diminishes as the time-horizon expands, which indicates there may be a point of diminishing returns when using workload estimates as inputs instead of physiological signals. Although, relying on workload estimates results in higher correlation coefficients. Thus, the workload estimate-based model performs better overall than using physiological signals solely in the general case.

**Table 5:** Accuracy comparison between a workload estimate-based and physiological signal-based performance prediction models.

**Model**	**Input type**	**MAE**	**Correlation**
*GM*_0_	Workload	0.111	0.329*
	Physiological	0.124	0.169*
*GM*_5_	Workload	0.112	0.326*
	Physiological	0.126	0.173*
*GM*_15_	Workload	0.114	0.299*
	Physiological	0.123	0.138*
*GM*_30_	Workload	0.116	0.294*
	Physiological	0.118	0.224*
*GM*_60_	Workload	0.117	0.274*
	Physiological	0.123	0.097*
*GM*_120_	Workload	0.124	0.168*
	Physiological	0.128	0.011
*GM*_300_	Workload	0.121	0.636*
	Physiological	0.128	-0.057*
*IM*_0_	Workload	0.093	0.622*
	Physiological	0.102	0.483*
*IM*_5_	Workload	0.096	0.624*
	Physiological	0.101	0.524*
*IM*_15_	Workload	0.094	0.617*
	Physiological	0.101	0.566*
*IM*_30_	Workload	0.097	0.617*
	Physiological	0.103	0.554*
*IM*_60_	Workload	0.095	0.274*
	Physiological	0.106	0.535*
*IM*_120_	Workload	0.098	0.613*
	Physiological	0.107	0.534
*IM*_300_	Workload	0.099	0.633*
	Physiological	0.109	0.556*

*indicates that the *p*-value is ≤ 0.

Both input types increased in accuracy (lower MAE, higher correlation) when using individual-specific data compared to their respective generalized models. A similar trend to the generalized models, when comparing the input types, is seen in the individual model results. The workload estimate-based model consistently outperforms the physiological based-model in terms of MAE and correlation coefficients. Additionally, there are no models for which the physiological based-model outperforms the workload estimate-based model. Thus, using workload estimates for performance prediction is better than using physiological signals for all of the analyzed time horizons when training on participant-specific data.

## 5. Discussion

Predicting future task performance accurately is a difficult and complex problem, but the two developed performance prediction models are a necessary step toward realizing intelligent machine systems that prevent future task performance decrements. It was predicted that the MAE values between the actual and generalized prediction model's performance values will be within 0.1, hypothesis **H**_**1**_. This hypothesis was partially supported for workload orderings **O_2_** and **O_3_**, but was not supported for **O_1_**. The generalized performance prediction model performs the best with **O_3_**, and the worst for **O_1_**, with **O_2_** having an intermediate performance. The discrepancy in performance can be attributed to (1) workload transitions, (2) the number of each workload condition present in the workload ordering, and (3) overall task performance calculation for the NASA MATB-II. **O_1_** had three underload workload conditions, where the resource management task was the primary contributor to the overall performance. If a participant experiences workload transitioning from overload to underload and the fuel tank levels were not maintained during the overload condition, the participant's overall performance drops quickly. If the participant pumps fuel at the fastest rate possible, there will still be a time delay until overall performance is optimal. This confound may attribute to lower prediction accuracy.

The ability to track performance trends will allow a system to trigger an adaptation to prevent a performance decrement. The second hypothesis (**H**_**2**_) evaluated the generalized performance prediction model's ability to track performance trends and was partially supported, due to the negative correlations for **O_1_**. There may be ordering confounds that hinder prediction accuracy or individual specific data is needed.

The individualized performance model results were better than the generalized models, as predicted by Hypotheses **H**_**3**_ and **H**_**4**_; except for **O_3_**'s underload condition. The two hypotheses were partially supported, which is attributed to an ordering effect. There was little difference between the generalized and individualized models' MAEs for **O_3_**'s underload condition; indicating that the models achieved similar accuracy. However, the generalized model tracked workload variations better, which indicates workload ordering had a negative effect on the individualized model. Overall, the individualized performance prediction model is better for intelligent human-machine system designers.

Hypothesis **H**_**5**_ predicted that the MAE values increase and the correlation coefficients decrease as the performance values are projected further into the future for both prediction models. This hypothesis was partially supported, as the average MAE difference between time horizons was marginal. However, there was a noticeable decrease in correlation coefficients. This result suggests that the developed models were able to maintain accuracy when predicting performance further into the future, but had more difficulty capturing the exact performance changes. Additionally, the IM models were more robust to increases in the time-horizon than the GM models, which means that intelligent human-machine systems can adapt to longer time-horizons when using an IM vs. a GM. It is expected that there is a limit to a model's predictive capabilities. For example, predicting task performance for an hour into the future will be inaccurate, but triggering an adaptation to prevent a potential performance decrement from occurring in an hour will likely be ineffective. Intelligent human-machine system designers will need to determine what timeframe is appropriate to prevent performance decrements from occurring due to system adaptations.

The remaining hypothesis (**H**_**6**_) stated that using workload estimates, rather than physiological-based features as inputs, will increase algorithm accuracy, due to the reduced input complexity. This hypothesis was fully supported for both the generalized and individualized models, which indicates that performance prediction models need to rely on workload estimates, rather than physiological signals only. This result is attributed to the workload assessment algorithm encoding more relevant information pertaining to task performance than the physiological signals, similar to feature reduction techniques used in machine-learning (e.g., principal component analysis).

Every human has a unique way of performing tasks that directly affects the overall task performance. Modeling individual human differences is challenging, but critical for mission success in intelligent human-machine teaming systems. Learning effects may also take place as a person becomes familiar with a new task or system. For example, participants tended to perform better on evaluation day 2 than they did on day 1 (Heard, 2019), due to learning effects. These effects have no impact on the generalized model, as it has no a priori information specific to a participant. It is unlikely that learning effects negatively impacted the individualized model results, due to including the first half of the participant's day 2 data when training. Future work will analyze learning effects that occurred in the evaluation and relate those to the predictive power of the individualized models.

The primary utility of the individualized model may be accounted for skill levels (e.g., novices, experts). All participants that completed the evaluation had no prior experience with the NASA MATB-II and were considered novices. This means certain patterns may have occurred in the data that the prediction models captured. Expert users will likely have less variance in their overall performance scores, which may make predicting future task performance easier for a generalized model. Having two separate general models (one for novices and one for experts) may lead to more accurate task performance predictions; however, such a split requires some a priori information about the human.

The individual performance prediction model can be improved through online learning, where the model is retrained/updated after every *n* minutes of interacting with an individual user using newly collected data. This approach biases the model toward the user and will lessen the model's population generalizability. Future work will compare the capabilities of the performance prediction model trained online with participant-specific data against the currently developed models.

The developed performance prediction model relied on the last three workload estimates to predict a single overall task performance value. More accurate predicted performance may be achieved by using additional workload estimates from the past. There may be a trade-off when using additional workload estimates, as incorporating outdated information may actually decrease accuracy. Future work will vary the number of past workload estimates in order to analyze the developed model's capabilities further.

Predicting overall task performance allowed for a single value prediction, which can be used to determine if an adaptation is needed. However, calculating overall task performance can be confounded by a wide-range of factors (i.e., multiple concurrent tasks, time delays). Higher accuracy may be achieved by predicting individual task performance values, rather than an aggregate value. The primary limitation to such an approach is the need for recognizing the human's current and future task focuses. For example, an adaptive NASA MATB-II system will need to predict which four tasks will be active in the next 3 min. A mission plan may provide some information (i.e., an automation hand-over is scheduled to occur), but spontaneous tasks (i.e., communications requests) will be difficult to predict. Future work will investigate predicting individual task performance values with a short-time horizon (i.e., 15 s), as an intelligent system may use this predicted information (e.g., reaction times) in a task scheduling paradigm.

## 6. Conclusion

Predicting future task performance accurately may allow machines to adapt their interactions with humans intelligently in order to prevent task performance decrements from occurring. Multi-dimensional workload estimates were used to create a generalized and an individualized performance prediction model to predict future performance across various time horizons. Both the generalized and individualized models were validated using data collected from the NASA MATB-II; a supervisory-based human-machine system. The results show that the individualized models were more accurate, but a generalized model may be viable in domains where individual-specific data is unavailable. The presented performance prediction models are a necessary step to realizing a human-machine teaming system that adapts autonomy levels and system interactions using generalized or individualized human-state information.

## Data availability statement

The raw data supporting the conclusions of this article will be made available by the authors, without undue reservation.

## Ethics statement

The studies involving human participants were reviewed and approved by the Vanderbilt University Institutional Review Board and the Oregon State University Institutional Review Board. The patients/participants provided their written informed consent to participate in this study.

## Author contributions

All authors listed have made a substantial, direct, and intellectual contribution to the work and approved it for publication.

## Funding

This work was supported by NASA Cooperative Agreement Number NNX16AB24A and by a Department of Defense Contract Number W81XWH-17-C-0252 from the CDMRP Defense Medical Research and Development Program.

## Conflict of interest

The authors declare that the research was conducted in the absence of any commercial or financial relationships that could be construed as a potential conflict of interest.

## Publisher's note

All claims expressed in this article are solely those of the authors and do not necessarily represent those of their affiliated organizations, or those of the publisher, the editors and the reviewers. Any product that may be evaluated in this article, or claim that may be made by its manufacturer, is not guaranteed or endorsed by the publisher.

## Footnotes

^1^Vanderbilt University IRB Approval Number #181741 and Oregon State University IRB Approval Number #8678.
